# Pulmonary Artery Pulsatility Index in Acute and Chronic Pulmonary Embolism

**DOI:** 10.3390/medicina61020363

**Published:** 2025-02-19

**Authors:** Mads Dam Lyhne, Eugene Yuriditsky, Vasileios Zochios, Simone Juel Dragsbaek, Jacob Valentin Hansen, Mads Jønsson Andersen, Søren Mellemkjær, Christopher Kabrhel, Asger Andersen

**Affiliations:** 1 Department of Clinical Medicine, Aarhus University, 8200 Aarhus N, Denmark; simone.jd@clin.au.dk (S.J.D.); jvh@clin.au.dk (J.V.H.); asgerandersen@gmail.com (A.A.); 2 Department of Anesthesiology and Intensive Care, Aarhus University Hospital, 8200 Aarhus N, Denmark; 3 Division of Cardiology, Department of Medicine, NYU Grossman School of Medicine, New York, NY 10016, USA; eugene.yuriditsky@nyulangone.org; 4 Department of Critical Care Medicine and ECMO, Glenfield Hospital, University Hospitals of Leicester NHS Trust, Leicester LE3 9QP, UK; vasileioszochios@doctors.org.uk; 5 Department of Cardiovascular Sciences, University of Leicester, Leicester LE3 9QP, UK; 6 Department of Cardiology, Aarhus University Hospital, 8200 Aarhus N, Denmark; andersen.mads@rm.dk (M.J.A.); soren.mellemkjaer@rm.dk (S.M.); 7 Department of Emergency Medicine, Massachusetts General Hospital, Boston, MA 02114, USA; ckabrhel@mgb.org; 8 Department of Emergency Medicine, Harvard Medical School, Boston, MA 02115, USA

**Keywords:** right heart catheterization, right ventricular function, acute pulmonary embolism, chronic thromboembolic pulmonary hypertension, pulmonary circulation, animal model

## Abstract

*Background and Objectives*: The pulmonary artery pulsatility index (PAPi) is an emerging marker of right ventricular (RV) injury but has not been well investigated in acute pulmonary embolism (PE) or chronic thromboembolic pulmonary hypertension (CTEPH). We aimed to investigate its discriminatory capabilities and ability to detect therapeutic effects in acute PE and CTEPH. *Materials and Methods*: This was a secondary analysis of data from both experimental studies of autologous PE and human studies of acute PE and CTEPH. PAPi was calculated and compared in (1) PE versus sham and (2) before and after interventions aimed at reducing RV afterload in PE and CTEPH. The correlations between PAPi, cardiac output, and RV to pulmonary artery coupling were investigated. *Results*: PAPi did not differ between animals with acute PE versus sham, nor was it affected by clot burden (*p =* 0.673) or at a 30-day follow-up (*p =* 0.242). Pulmonary vasodilatation with oxygen was associated with a reduction in PAPi (4.9 [3.7–7.8] vs. 4.0 [3.2–5.6], *p* = 0.016), whereas positive inotropes increased PAPi in the experimental PE. In humans, PAPi did not change consistently with interventions. Balloon pulmonary angioplasty did not significantly increase PAPi (6.5 [4.3–10.7] vs. 9.8 [6.8–14.2], *p* = 0.1) in patients with CTEPH, and a non-significant reduction in PAPi (4.3 ± 1.6 vs. 3.3 ± 1.2, *p* = 0.074) was observed in patients with acute PE who received sildenafil. PAPi did not correlate well with cardiac output or measures of RV to pulmonary artery coupling in either species. *Conclusions*: PAPi did not detect acute, experimental PE or changes as a result of therapeutic interventions in patients with hemodynamically stable acute PE or CTEPH. However, it did change with pharmacological interventions in the experimental PE. Further research should establish its utility in detecting and monitoring RV injury in different clinical phenotypes of acute PE and CTEPH.

## 1. Introduction

Acute pulmonary embolism (PE) is a potentially fatal condition that may result in chronic thromboembolic pulmonary hypertension (CTEPH) despite appropriate therapies. Both acute PE and CTEPH are characterized by elevated right ventricular (RV) afterload and may progress to RV injury (from RV dilatation to failure) and eventually cause death [1].

In both conditions, the RV compensates to the increased afterload through homeometric adaptation, increasing intrinsic contractility, as well as heterometric adaptation, causing RV dilatation, exploiting the Starling forces to preserve the flow. The RV will compensate by generating higher pulmonary artery pressures (PAPs) and maintaining cardiac output to meet the metabolic demand. However, if the afterload exceeds the RV’s compensatory mechanisms, the RV will become dysfunctional, which may lead to decreasing PAP and cardiac output. Simultaneously, RV dysfunction with reduced ventricular emptying will usually cause an increase in right atrial pressure (RAP) [2,3].

The pulmonary artery pulsatility index (PAPi) combines the PAP and RAP and is calculated as follows:

PAPi = PAPP/RAP = (PASP − PADP)/RAP,(1)

where PAPP is the pulmonary artery pulse pressure defined as the difference between the pulmonary artery systolic pressure (PASP) and the pulmonary artery diastolic pressure (PADP). The PAPi is calculated from three pressure measurements but is susceptible to changes in RV stroke volume (SV), pulmonary vascular resistance (PVR), and pulmonary artery capacitance (PAC) [4,5]. PAPi is an emerging marker for RV injury in acute critical care [6,7] but has primarily been studied in the context of predicting RV injury in patients with advanced left heart failure who are referred for heart transplantation or left ventricular assist device implantation [8,9]. Few studies have investigated its utility in isolated RV involvement due to increased afterload such as in acute PE and CTEPH [10,11].

The current study aimed to investigate the discriminatory utility of PAPi in acute PE and the index’s capability to detect the physiological effects of therapeutic interventions in acute PE and CTEPH.

## 2. Materials and Methods

### 2.1. Study Design and Data

This was a post hoc analysis of previously published studies on acute PE and CTEPH in both experimental (total n = 58) [12,13,14,15] and clinical settings (total n = 140) [16,17]. All the studies describe either the natural history of acute PE compared to sham, or an intervention aimed to reduce RV afterload and/or improve RV function in acute PE or CTEPH.

**Figure 1:**
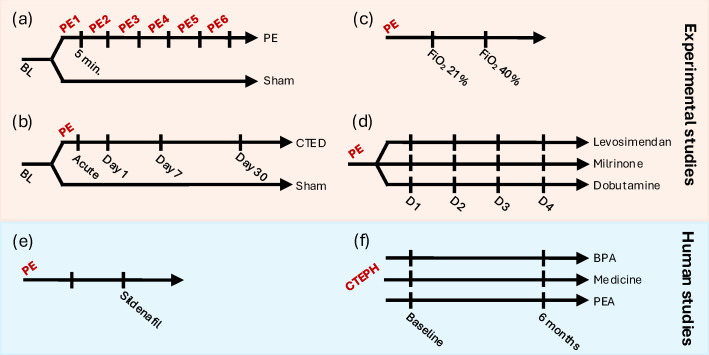
Study designs of included studies. (**a**) Animals were randomized to six consecutive PE inductions or sham with evaluations performed at baseline and 5 min after each induction [13]. (**b**) Animals were randomized to acute PE or sham with evaluations performed at BL and after 1, 7, and 30 days to induce CTED [12]. (**c**) Animals with PE received increased FiO_2_ to reduce pulmonary pressure [14]. (**d**) Animals with PE were randomized to four escalating doses (D1–4) of three different inodilators [15]. (**e**) Patients with acute, intermediate-risk PE were evaluated before and after treatment with sildenafil [16]. (**f**) Patients with CTEPH were evaluated at baseline and 6 months following either balloon pulmonary angioplasty, pulmonary endarterectomy, or pharmacological treatment only [17]. Abbreviations: BL, baseline; CTED, chronic thromboembolism pulmonary disease; CTEPH, chronic thromboembolic pulmonary hypertension; D, dose; FiO_2_, fraction of inspired oxygen; PE, pulmonary embolism; PEA, pulmonary endarterectomy.

Of the experimental studies, these included data from two prospective, randomized, controlled, experimental studies comparing autologous PE versus sham either immediately (five minutes after each consecutive PE) [13] (Figure 1a) or at a 30-day follow-up aimed to induce chronic thromboembolic pulmonary disease (CTED) [12] (Figure 1b); one prospective, observational, experimental study where animals with PE received supplemental oxygen to lower RV afterload [14] (Figure 1c); and one randomized, controlled, experimental study where animals with PE were randomized to receive different inodilators [15] (Figure 1d). All the experimental studies were based on the same model of autologous PE and inclusion criteria; see details below.

Of the human studies, these included data from one prospective, clinical, randomized, controlled trial where patients with PE were randomized to receive a phosphodiesterase-5 inhibitor or placebo [16] (Figure 1e); and one clinical, retrospective observational study where patients with CTEPH underwent pulmonary endarterectomy, balloon pulmonary angioplasty, or pharmacological treatment only [17] (Figure 1f). See details below.

We reviewed the study data and calculated the PAPi [5]. Data on baseline cardiac output (CO) and measures of right ventricular–pulmonary artery (RV-PA) coupling were extracted. For the experimental studies, we used the RV end-systolic elastance/arterial elastance ratio (Ees/Ea) obtained at the same timepoints from invasive pressure–volume (PV) loop measurements to calculate RV-PA coupling [18]. In the clinical CTEPH study, the tricuspid annular plane systolic excursion (TAPSE) to PASP (TAPSE/PASP) ratio was used as a non-invasive transthoracic echocardiographic (TTE) surrogate of RV-PA coupling [19].

### 2.2. Ethics

All the experimental studies were performed according to the ARRIVE guidelines on animal research [20] and were approved by the Danish Animal Research Inspectorate (licenses no. 2018-15-0201-0152, 2023-15-0201-01451 and 2016-15-0201-00840). All the animals were handled in accordance with Danish standards of proper animal care. All the researchers had a FELASA license to conduct animal research.

The clinical retrospective study was approved by the Central Denmark Region Ethical Board (license no. 1–45–70-93-21) and the local institutional administration. The clinical randomized controlled trial was registered on ClinicalTrials.gov (NCT04283240) and approved by the regional ethics committee.

### 2.3. Experimental Studies

The detailed protocols and procedures have been described previously [13,14]. All the animals underwent right heart catheterization (RHC), which enabled the measurement of PASP, PADP, and RAP. A right ventricular PV catheter was inserted. Blood was drawn to create large, autologous PEs, which were re-introduced to the central venous circulation to cause central PE.

In two studies comparing PE with sham, animals were randomized to receive consecutive PE or sham/saline infusion until mean pulmonary arterial pressure (mPAP) had doubled from baseline, or a similar number of saline infusions. In one study, hemodynamic evaluation was performed five minutes after each consecutive infusion to relate to clot burden [13], whereas in the other study, hemodynamic evaluations were repeated at 30 min and again at 1, 7, and 30 days after PE induction to induce CTED [12] (Figure 1a,b).

In the two pharmacological interventional studies, animals also received PEs until mPAP was doubled from baseline. In one study, animals were subjected to an increased fraction of inspired oxygen (from 21% to 40%) to cause pulmonary vasodilatation and RV afterload reduction with the evaluation performed after 15 min [14] (Figure 1c). In the other study, animals with PE were randomized to three different inodilators (four escalating doses of either levosimendan [loading dose of 2.4, 6, 24, and 60 mg/kg], milrinone [loading dose of 10, 30, 100, and 300 mg/kg], or dobutamine [0.1, 0.3, 1, and 3 mg/kg/min]) to improve RV function which was evaluated 30 min after each dose [15] (Figure 1d).

### 2.4. Human Studies

In the human randomized controlled trial, patients (n = 20) with acute intermediate-high risk PE [1] were randomized 1:1 to receive a single dose of a phosphodiesterase-5 inhibitor (sildenafil, 50 mg) or placebo. RHC and TTE were performed before and immediately (0.5–1.5 h) after randomization [16]. This study included the ten patients randomized to sildenafil, and data for PAPi calculation were available for n = 7 (70%). TTEs were performed according to guidelines [21,22] by trained cardiologists.

At our national center for interventional CTEPH treatment, 130 patients were analyzed with RHC and TTE before and after either balloon pulmonary angioplasty (n = 54), surgical pulmonary endarterectomy (n = 63), or medical treatment only (n = 13). Follow-up took place at 6 months. TTE and RHC were performed within two days. The TTE images were analyzed and reported retrospectively with the observers blinded to the data source [17].

### 2.5. Statistical Analysis

No a priori power calculation was performed since this was a secondary analysis of previously published data. Data were evaluated for normal distribution by the Shapiro–Wilk test. Data are presented as mean ± SD if normally distributed and median [IQR] if not. Data with two timepoints were compared by a paired *t*-test or Wilcoxon signed-rank test where appropriate. Data with three or more timepoints were compared by one-way or two-way ANOVA where appropriate with control for multiple comparisons by Dunnett’s, Sidak’s, or Dunn’s test, respectively. Correlations between PAPi and CO or TAPSE/PASP were analyzed by linear regression. GraphPad Prism version 10.0.0 (GraphPad Software, Boston, MA, USA) or RStudio, ver. 2023.06.0 were used for the analyses. A *p*-value < 0.05 was considered statistically significant.

## 3. Results

### 3.1. Experimental Results

**Figure 2:**
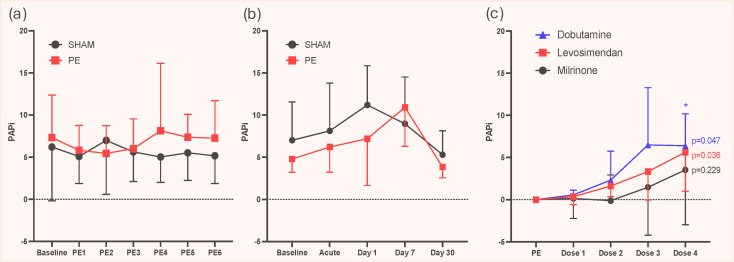
Pulmonary artery pulsatility index (PAPi) in the experimental PE. (**a**) Changes in PAPi five minutes after the induction of consecutive, autologous PE (PE1–6) or sham procedure with saline infusion. (**b**) Changes in PAPi after the induction of acute PE and after 1-, 7-, and 30-day follow-up. (**c**) Compared to PAPi after PE induction, the inodilators levosimendan and dobutamine (but not milrinone) increased PAPi in a dose-dependent manner. Abbreviations: PAPi, pulmonary artery pulsatility index; PE, pulmonary embolism.

In the study comparing autologous, acute PE to sham within minutes after the induction, PAPi did not differ (*p* = 0.673, Figure 2a). We noted that PASP and PADP increased compared to sham (*p* < 0.001 for both) but to a similar extent, yielding an unaltered PAPP. RAP (*p* = 0.702) and CO did not differ between the groups either which further explains the neutral effects on PAPi.

In the long-term study, PAPi also did not differ between PE and sham animals during the 30-day follow-up (*p* = 0.242, Figure 2b). The neutral finding was explained by an increase in both PASP (54 ± 8 vs. 29 ± 5 mmHg, adjusted *p* = 0.009) and PADP (24 ± 5 vs. 11 ± 3 mmHg, adjusted *p* = 0.002) in the acute phase after PE induction but with a concomitant increase in RAP (6 ± 2 vs. 3 ± 1 mmHg, ANOVA *p* = 0.0064, adjusted *p* = 0.121).

RV afterload reduction with increased oxygen supply resulted in a reduction in PAPi (4.9 [3.7–7.8] vs. 4.0 [3.2–5.6], *p* = 0.016). This was driven by a reduction in pulmonary pressures and no changes in RAP.

Levosimendan and dobutamine, but not milrinone, induced an increase in PAPi with a dose–response relationship (Figure 2c).

**Figure 3:**
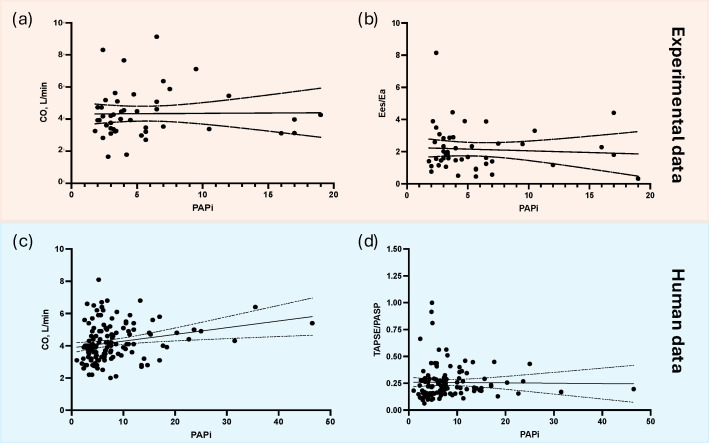
Correlation between PAPi and CO (**a**,**c**) or measures of right ventricular–pulmonary artery coupling (**b**,**d**). See text for details. Baseline measures of CO and PAPi (**a**) and baseline right ventricular Ees/Ea (derived from invasive pressure–volume loop acquisition) and PAPi (**b**) from the included experimental studies, n = 46. Baseline measures of CO and PAPi (**c**) and baseline measures TAPSE/PASP (derived from transthoracic echocardiography) and PAPi (**d**) in patients with CTEPH, n = 130. Abbreviations: CO, cardiac output; CTEPH, chronic thromboembolic pulmonary hypertension; Ea, arterial elastance; Ees, end-systolic elastance; PAPi, pulmonary artery pulsatility index; PASP, pulmonary artery systolic pressure; TAPSE, tricuspid annular plane systolic excursion.

PAPi did not correlate with RV-PA coupling (Spearman coefficient −0.085, *p* = 0.574, slope −0.021 [−0.120–0.077], r^2^ = 0.004) nor CO (Spearman coefficient 0.097, *p* = 0.520, slope 4 [−104–113], r^2^ = 0.0001, Figure 3a,b).

### 3.2. Human Results

Patient characteristics from the two human studies have been reported previously [16,17] and important characteristics are summarized in Table 1.

Sildenafil treatment in acute PE resulted in a non-significant reduction in PAPi (4.3 ± 1.6 vs. 3.3 ± 1.2, *p* = 0.074). Sildenafil did not change PASP, PADP, PVR, or SV and did, thereby, not successfully reduce RV afterload [16].

In patients with CTEPH, baseline PAPi for all the patients was 6.7 [4.1–10.9]. Neither balloon pulmonary angioplasty (6.5 [4.3–10.7] vs. 9.8 [6.8–14.2], *p* = 0.1), pulmonary endarterectomy (6.3 [4.0–8.3] vs. 7.0 [3.6–13.0], *p* = 0.92), nor medical treatment (5.4 [4.0–8.8] vs. 3.6 [3.5–3.6]) changed PAPi significantly. Balloon pulmonary angioplasty did reduce RAP, PASP, and PVR but did not alter CI, which can explain the neutral finding. The same was observed for pulmonary endarterectomy patients, who also displayed improved CI [17].

PAPi did not correlate with a measure of RV-PA coupling (TAPSE/PASP; Spearman coefficient 0.15, *p* = 0.1, slope −0.0003 [−0.0048; 0.0041], r^2^ = 0.0002) but did statistically correlate with CO (Spearman coefficient 0.29, *p* = 0.0005, slope 0.042 [0.012; 0.071], r^2^ = 0.05) among patients with CTEPH (Figure 3).

## 4. Discussion

The present post hoc study analyzed the PAPi in the experimental and human studies of acute PE and CTEPH. In our experimental model, the PAPi did not distinguish between the experimental PE and sham in the acute or prolonged phase but did change with afterload reduction or positive inotropic support. In humans, the PAPi did not change significantly following interventions. In both species, the PAPi did not correlate convincingly with CO or measures of RV-PA coupling. In humans, though, we used the TAPSE/PASP ratio as a surrogate marker for RV-PA coupling as it is clinically relevant and more available than gold-standard invasive pressure–volume loop measures (Figure 3, Table 2).

The PAPi is an emerging marker of RV injury and clinical deterioration in a broad selection of diseases and has been studied in populations with acute left ventricular failure requiring a left ventricular assist device [5,8,23,24]. In acute left ventricular failure, pulmonary congestion increases PVR and left ventricular filling pressure. The difference between left ventricular end-diastolic pressure, pulmonary arterial wedge pressure, and PADP is negligible, and acute left ventricular failure causes post-capillary pulmonary hypertension as long as the RV compensates. The increased PADP will reduce the PAPP (PAPi numerator) and disproportionately lower PAPi more than that reflected in the RV function alone [24]. With a dysfunctional RV, the RAP increases, and pulmonary artery pressures decrease, yielding a low PAPi [5]. The PAPi has not been sufficiently investigated in PE or CTEPH affecting the RV.

### 4.1. Acute Pulmonary Embolism

We found that PASP, PADP, and RAP changed concomitantly in the experimental PE, yielding no changes in PAPi (Figure 2a,b). The lack of discriminatory power of PAPi in acute PE may be explained by the complex pathophysiology of acute PE, where the release of several neuro-humoral factors affects pulmonary vascular tone, inducing pulmonary vasoconstriction and thereby altering PAC [1,25]. PAPi is very susceptible to changes in SV and PAC. PAPP is defined as PAPP = RV SV/PAC, and, accordingly, PAPi increases with lower PAC but also increases with higher SV. However, the effects of SV changes are dependent on PAC or RAP [5]. Interventions improving both PAC and SV may lead to a neutral effect on PAPi, and interventions affecting pulmonary vasculature may increase PAC and lower PAPP, whereas interventions improving RV SV increase PAPP. In acute PE, PE-induced pulmonary vasoconstriction will decrease PAC whereas PE-induced RV injury will lower SV resulting in an unchanged PAPP and thereby PAPi. This analysis is restricted to cardiovascular changes since external factors on the lung parenchyma or thoracic cavity may also affect PAC, SV, and thereby PAPi. Accordingly, such complex right heart pulmonary circulation interactions may potentially limit the use of PAPi in acute PE (Table 2).

The effects of positive inotropes and vasodilators on PAPi have been identified as a knowledge gap [5]. This study showed that some inodilators could increase PAPi in acute experimental PE in a dose–response manner (Figure 2), whereas pulmonary vasodilatation by increased oxygen supply reduced PAPi, which would be interpreted as a deterioration. However, this was driven by lower pulmonary artery pressures which is interpreted as an improvement. Accordingly, changes in PAPi alone must be interpreted with caution but may have a role as part of a multi-modal pulmonary hemodynamic monitoring and diagnostic approach in patients at risk of pulmonary vascular dysfunction and RV injury.

Our neutral findings may question the clinical applicability of PAPi, but PAPi could still serve as a secondary marker in subsets of patients. The limited utility of PAPi may be restricted to compensatory or stable RV states. PAPi combines RV forward pressure generation with backward pressure, i.e., it maintains a high value as long as the RV adapts to increased afterload [4,5]. This may explain our neutral findings since the experimental model reflects non-high-risk PE with maintained CO and mean systemic arterial pressure [1]. We speculate that the PAPi might function as a secondary marker by decreasing high-risk acute PE characterized by systemic hypotension when the RV fails to meet flow demand. However, it is difficult to predict since in severe RV injury (e.g., RV failure states), RAP (PAPi denominator) is usually elevated, concomitant with the myocardial inability to generate high pulmonary arterial pressures (PAPi numerator). High-risk PE is defined solely by low systemic pressure [1], and PAPi may discriminate if systemic hypotension is caused by PE-induced RV injury or not. Further research on PAPi in acute (high-risk) PE is warranted and future directions should include studies to determine the predictive value and optimal cutoff of PAPi to predict adverse outcomes in intermediate- and high-risk PE and compare its utility to other non-invasive metrics, and, secondly, investigate whether PAPi can be used prospectively to guide clinical decision making and RV-targeted therapies. Such studies may be possible with the growing use of catheter-directed therapies in intermediate- and high-risk patients, where peri-procedure invasive pressure measurements may be feasible.

### 4.2. Chronic Thromboembolic Pulmonary Hypertension

In our animal model of autologous CTED [12], PAPi did not change over 30 days of follow-up. This may be due to a high degree of vascular autoregulation in the animals, where PVR and pulmonary artery pressure normalized in the early days after PE induction. Accordingly, the animals did not develop CTEPH [12].

In patients with CTEPH, we found that different treatments did not significantly alter PAPi, though balloon pulmonary angioplasty tended to increase the index. The mechanical CTEPH-specific interventions did reduce PVR, but given the concomitant changes in the other determinants of PAPi (i.e., PAC, SV, and RAP), PAPi did not change [5,17]. Our observations are supported by the study by Sławek-Szmyt et al. [26] where PAPi did increase following combined CTEPH-specific pharmacological interventions and balloon pulmonary angioplasty. Our study conflicts with the observations from Martin-Suarez et al. [11] who found significant PAPi reduction, but this occurred on the first postoperative day following pulmonary endarterectomy. This was caused by a reduction in pulmonary pressure but no changes in RAP or CO. We observed significant RAP reduction and CO increase in our population but this occurred at follow-up months later, making it meaningless to directly compare [11,17]. Waziri et al. [27] did not calculate PAPi but showed that pulmonary endarterectomy led to an increase in PAC and reduced PVR, RAP, and pulmonary pressures, but did not change SV. Accordingly, the procedural change in PAPi is difficult to predict. The interpretation of PAPi following pulmonary endarterectomy is even more complex due to changes in RV function caused by sternotomy and cardiac surgery alone which may affect RV contractile pattern and may influence SV and pressure generation [28,29].

As mentioned, a PAPi reduction would be interpreted as a deterioration. PAPi has previously been associated with outcomes in patients with CTEPH or pulmonary hypertension from other causes [10,11,26,30], but these associations were based on baseline PAPi measurements [11,26]. Mechanical CTEPH interventions improve patient outcomes [29,31], and a PAPi increase would be expected to improve clinical outcomes, but this association has yet to be investigated.

The median value of PAPi among patients with CTEPH in a compensatory state undergoing elective procedures in our study was comparable to other studies on patients with CTEPH or other categories of pulmonary hypertension [10,11,27,31]. A high PAPi in these studies was driven by elevated pulmonary artery pressures, i.e., a compensatory state. When PAPi was low in different subgroup analyses, this was driven by elevated RAP values as a sign of early decompensation rather than reduced PAP values [10,11,26,30]. Patients with pulmonary hypertension show higher PAPi values than other acute conditions such as acute left ventricular failure or the need for a left ventricular assist device [8,9,24]. The differences can be explained by different disease etiologies and the speed of disease progression, as the RV may adapt to extremely high PVR over time [2,3].

**Table 2:** Strengths and limitations of PAPi.

Strengths	Limitations
- Indicator of right ventricular function [5]	- Relies on invasive pressure measurements
- Associated with outcome and right ventricular injury in patients with advanced left heart failure [8,9,24]	- Susceptible to changes in stroke volume, pulmonary arterial capacitance, pulmonary vascular resistance, and pulmonary arterial wedge pressure [5]
- Associated with outcome in pulmonary arterial hypertension and chronic thromboembolic pulmonary hypertension [10,11,26,30]	- Right atrial pressure is influenced by both cardiac function and venous return (ie stressed venous volume and venous compliance/constriction) [5]
**Results from the present study**	
- Detects changes in right ventricular afterload in experimental acute pulmonary embolism	- Not correlated with measures of cardiac output or right ventricular to pulmonary artery coupling
- Detects changes from positive inotropy in experimental acute pulmonary embolism	- Does not change at long-term follow-up after mechanical intervention in chronic thromboembolic pulmonary hypertension
	- Does not distinguish between experimental, stable acute pulmonary embolism and sham

Strengths and limitations of PAPi from the previous and present studies. See text for further details.

### 4.3. Limitations

This study has a number of limitations to consider. First, this was a post hoc analysis of previously conducted studies neither designed nor powered for PAPi measurements, and heterogenous data may have been combined. Secondly, a comparison between porcine and human data must be carried out with caution. Patients with PE and CTEPH are typically older and comorbid, whereas pigs used for laboratory investigations are younger and healthy. Changes in PAPi with PE induction and therapeutic interventions may not be similar across species. Third, we used the echocardiographic ratio TAPSE/PASP as a non-invasive metric for RV-PA coupling, which has been validated against invasive PV measurements in patients with pulmonary hypertension, but the utility of the ratio is still in debate [19,32]. We did not have hemodynamic data for those very few patients experiencing reperfusion lung injury post-procedure, which could be investigated in future studies. Lastly, our neutral observations do not necessarily imply no clinical relevance of the PAPi but that PAPi should be interpreted as part of a multi-modal monitoring and diagnostic approach. Our study is limited to hemodynamically stable subjects, and PAPi may be more useful in decompensated states.

## 5. Conclusions

Our study showed that PAPi was not useful in the detection of acute, experimental PE. PAPi did not change by therapeutic interventions in patients with hemodynamically stable acute PE or CTEPH. However, it did change with RV afterload reduction and positive inotropic support in experimental, acute PE. Further research is warranted to establish its utility across the spectrum of RV injury (RV dilatation/dysfunction/failure) and pulmonary vascular dysfunction in different phenotypes of pulmonary thromboembolism.

## Figures and Tables

**Table 1:** Patient characteristics.

	Acute PE (n = 10) [16]	CTEPH (n = 130) [17]
Age, years	63 ± 9	70 [59–75]
Sex, male	7 (70%)	72 (52%)
BMI, kg/m^2^	30 ± 4	27 [24–31]
COPD	2 (20%)	37 (27%)
Smoking	10 (100%)	83 (60%)
Previous VTE	1 (10%)	129 (93%)
Heart rate, bpm	85 ± 12	78 [70–87]
Systolic arterial pressure, mmHg	137 ± 24	131 [119–148]
Right heart catheterization		
RAP, mmHg	7 ± 2	8 [5–11]
mPAP, mmHg	27 ± 4	40 ± 10
PASP, mmHg	47 ± 8	77 ± 19
PADP, mmHg	17 ± 3	27 ± 8
PVR, WU	2.3 ± 1.0	8.4 [5.6–11.8]
Transthoracic echocardiography		
TAPSE, mm	17 ± 4	18 ± 5
TRG, m/s	2.9 ± 1.4	4.1 ± 0.7
RV/LV	1.1 ± 0.1	1.2 ± 0.3
RV FAC, %	34 ± 15	25 ± 12

Baseline patient characteristics from the previously published studies [16,17] were used for the calculation of PAPi in the present study. Abbreviations: BMI, body mass index; COPD, chronic obstructive pulmonary disease; CTEPH, chronic thromboembolic pulmonary hypertension; FAC, fractional area change; mPAP, mean pulmonary arterial pressure; PADP, pulmonary arterial diastolic pressure; PASP, pulmonary arterial systolic pressure; PE, pulmonary embolism; PVR, pulmonary vascular resistance; RAP, right atrial pressure; RV/LV, right ventricular to left ventricular diameter ratio; TAPSE, tricuspid annular plane systolic excursion; TRG, tricuspid regurgitation gradient; VTE, venous thromboembolism; WU, Wood Unit.

## Data Availability

Data from experimental studies are publicly accessible through FigShare (https://doi.org/10.6084/m9.figshare.28262942, accessed on 23 January 2025). Data from human studies will be available upon reasonable request to the corresponding author due to ethical reasons.
